# blind or schizophrenic but not both

**DOI:** 10.1192/j.eurpsy.2022.2043

**Published:** 2022-09-01

**Authors:** C. Fatima Zahra, I. Katir, A. Korchi, S. Belbachir, A. Ouanass

**Affiliations:** CHU Avicenne Rabat, Psychiatric Hospital Ar -razi Salé, salé, Morocco

**Keywords:** congenital, blindness, schizophrénia, protect

## Abstract

**Introduction:**

Although visual impairment appears to be a risk factor for schizophrenia, early blindness may be protective , It’s a phenomenon that has puzzled even the smartest scientific brains for decades. It might surprise you: no person born blind has ever been diagnosed with schizophrenia.

**Objectives:**

The objective of this research is to discover the relationship between schizophrenia and congenital blindness is there a protective gene ! is that visual perception constitutes an essential stage in the onset of the disease itself !

**Methods:**

Case study of a family consisting of thirteen brothers and sisters, three of whom were blind at birth, three with schizophrenia. the study of the files of schizophrenic patients hospitalized in our structure since it opened in the 1970s

**Results:**

Case study of a family consisting of thirteen brothers and sisters, three of whom were blind at birth, three with schizophrenia, but there is none with blindness at birth and schizophrenia. PLus on the basis of medicals files there is no case of schizophrenia with blindness at birth. Preliminary observational analysis of this clinical case suggests the following hypothesis: the presumed protective role of congenital blindness against schizophrenia. The bibliographic research has objectified three recent studies in this direction in Australia, Denmark, and the USA.

**Conclusions:**

The relationship between schizophrenia and congenital blindness is still unrecognized and controversial

**Several studies are done in this direction, but so far there is no assertion or confirmation of the hypothesis**

**Disclosure:**

No significant relationships.

